# Mfsd2a (Major Facilitator Superfamily Domain Containing 2a) Attenuates Intracerebral Hemorrhage–Induced Blood–Brain Barrier Disruption by Inhibiting Vesicular Transcytosis

**DOI:** 10.1161/JAHA.117.005811

**Published:** 2017-07-19

**Authors:** Yuan‐Rui Yang, Xiao‐Yi Xiong, Juan Liu, Li‐Rong Wu, Qi Zhong, Kai Zhou, Zhao‐You Meng, Liang Liu, Fa‐Xiang Wang, Qiu‐Wen Gong, Mao‐Fan Liao, Chun‐Mei Duan, Jie Li, Mei‐Hua Yang, Qin Zhang, Chang‐Xiong Gong, Qing‐Wu Yang

**Affiliations:** ^1^ Department of Neurology Xinqiao Hospital The Third Military Medical University Chongqing China

**Keywords:** blood–brain barrier, intracerebral hemorrhage, Mfsd2a, vesicular transcytosis, Blood-Brain Barrier, Cerebrovascular Disease/Stroke, Intracranial Hemorrhage

## Abstract

**Background:**

Blood–brain barrier (BBB) disruption aggravates brain injury induced by intracerebral hemorrhage (ICH); however, the mechanisms of BBB damage caused by ICH remain elusive. Mfsd2a (major facilitator superfamily domain containing 2a) has been known to play an essential role in BBB formation and function. In this study, we investigated the role and underlying mechanisms of Mfsd2a in BBB permeability regulation after ICH.

**Methods and Results:**

Using ICH models, we found that Mfsd2a protein expression in perihematomal brain tissues was significantly decreased after ICH. Knockdown and knockout of Mfsd2a in mice markedly increased BBB permeability, neurological deficit score, and brain water contents after ICH, and these were rescued by overexpressing Mfsd2a in perihematomas. Moreover, we found that Mfsd2a regulation of BBB permeability after ICH correlated with changes in vesicle number. Expression profiling of tight junction proteins showed no differences in Mfsd2a knockdown, Mfsd2a knockout, and Mfsd2a overexpression mice. However, using electron microscopy following ICH, we observed a significant increase in pinocytotic vesicle number in Mfsd2a knockout mice and decreased the number of pinocytotic vesicles in mouse brains with Mfsd2a overexpression. Finally, using multiple reaction monitoring, we screened out 3 vesicle trafficking–related proteins (Srgap2, Stx7, and Sec22b) from 31 vesicle trafficking‐related proteins that were markedly upregulated in Mfsd2a knockout mice compared with controls after ICH.

**Conclusions:**

In summary, our results suggest that Mfsd2a may protect against BBB injury by inhibiting vesicular transcytosis following ICH.

**Clinical Perspective**

**What Is New?**

- This study proposes that the mechanism underlying the important role of Mfsd2a in decreasing blood–brain barrier permeability is inhibition of vesicular transcytosis of brain microvascular endothelial cells after intracerebral hemorrhage, which has implications for brain homeostasis.

**What Are the Clinical Implications?**

- Our findings suggest that increased Mfsd2a protein expression may provide a novel therapeutic target for improving blood–brain barrier permeability to treat intracerebral hemorrhage.

## Introduction

Intracerebral hemorrhage (ICH) is a common and fatal subtype of stroke that is induced by bleeding in small blood vessels within the cerebral parenchyma and subsequent hematomas.^1^ Brain injury caused by ICH can result in high rates of mortality and morbidity, which currently represents a serious threat to public health. Although considerable progress has been made in animal and preclinical studies, effective therapeutic strategies for ICH are still lacking.^2^, ^3^

Blood–brain barrier (BBB) dysfunction is believed to be a hallmark of ICH‐induced brain injury,^1^ which can be caused by many pathogenic factors. Previous studies reported that blood components and/or metabolites, such as thrombin, fibrin, and erythrocytes, acutely injure the BBB, causing ICH‐induced BBB hyperpermeability.^4^, ^5^, ^6^ Furthermore, this perihematomal leukocyte infiltration and microglia activation can induce an inflammatory response in the perihematomal area that accelerates the ICH‐induced BBB injury.^7^, ^8^ In addition, many other elements within the inflammatory response, such as monocytes, cytokines, and matrix metalloproteinases, can alter BBB function in ICH.^3^ Taken together, these studies showed that the BBB injury exhibited increased permeability, which is an important molecular and cellular event that further aggravates brain injury after ICH.^6^ Theoretically, BBB permeability is strictly regulated by the paracellular pathway (alterations in tight junction function) and/or the transcellular route (vesicular transcytosis).^9^, ^10^ Previous studies reported that ICH‐induced BBB is regulated by the tight junction network via the paracellular route, but the transcellular route has long been ignored.^11^, ^12^ Consequently, the role of vesicular transcytosis in brain injury after ICH still needs to be investigated.

Mfsd2a (major facilitator superfamily domain containing 2a, also known as sodium‐dependent lysophosphatidylcholine symporter 1) is a transmembrane protein.^13^, ^14^ Recent studies indicate that Mfsd2a regulates nutrient supply from the blood into the brain while mediating BBB integrity.^13^, ^15^ A previous study identified Mfsd2a as a key regulator of BBB function. Mfsd2a is selectively expressed in the blood vessel endothelium and may suppress vesicular transcytosis in endothelial cells in the central nervous system.^16^ Overall, Mfsd2a plays a very important role in BBB function, and it remains unclear if Mfsd2a plays a role in BBB disruption after ICH.

In the current study, we investigated the role of Mfsd2a in BBB injury induced by ICH. Our results indicate that selectively expressing endothelial Mfsd2a suppresses ICH‐induced BBB disruption by inhibiting vesicular transcytosis.

## Materials and Methods

### Animals

A total of 200 male C57BL/6 mice (aged 8–10 weeks, 22–26 g) were purchased from the Animal Center of the Third Military Medical University (Chongqing, China). Mfsd2a knockout (Mfsd2a^−/−^) mice were generated by Beijing Biocytogen Co., Ltd (Beijing, China). The mice were housed in an environment with a 12‐hour light/dark cycle and ad libitum access to food and water throughout the experimental period. All experimental protocols were approved by the animal management committee of the Third Military Medical University. The mice were randomly divided into groups, and the investigators were blinded to the groups and genotypes of the mice.

### Generation of Mfsd2a^−/−^ Mice

Mfsd2a^−/−^ mice were generated by nonhomology end joining, which is induced by 2 double‐strand break repairs after introduction of 2 single‐guide RNAs with Cas9.^17^ Two single‐guide RNAs were designed to target a region upstream of exon 3 and downstream of exon 13, respectively. Different concentrations of Cas9 mRNA and single‐guide RNAs were mixed and coinjected into the cytoplasm of 1‐cell‐stage fertilized eggs to generate chimeras. Polymerase chain reaction genotyping and sequencing revealed that some pups carried deletions of about 10 kb spanning 2 single‐guide RNA target sites, removing the Mfsd2a exon 3 to 13 (Figure S1A and S1B). Genotyping of mutant mice was performed by Western blot and immunofluorescence staining.

### ICH Model

Establishment of the ICH model was described previously.^18^ Briefly, mice were anesthetized with 4% chloral hydrate (400 mg/kg) and fixed on a mouse stereotactic apparatus. Autologous whole blood (20 μL) was collected from the mouse tail vein and then injected into the striatum without anticoagulant at 2 μL/min through a stereotactic apparatus at 0.8 mm anterior and 2 mm left lateral to the bregma and at a depth of 3.5 mm. The needle was held in place for another 10 minutes until the blood coagulated, and then the microinjector was slowly pulled out. The skull was sealed with bone wax, and the wound was closed by sutures. Rectal temperature was maintained at 37°C throughout the procedure. In parallel, sham mice were subjected to the same manipulations except that 20 μL sterile saline was administered into the left striatum. The success rate of the model was 95%. Failed models and deceased mice were excluded from this study.

### Brain Tissue Preparation

Brains were extracted and immediately placed on ice when the animals were euthanized. A total of 5 mm of brain tissue surrounding the hematoma was collected for further analysis (Figure S2A).

### Measurement of BBB Permeability

BBB permeability was measured using Evans blue dye (EB) extravasation technique, as described previously.^19^ Briefly, mice were injected intravenously with 2% EB (4 mL/kg) in sterile saline at each time point. Three hours later, mice were deeply anesthetized and perfused with 50 mL heparinized saline through the left ventricle for 15 minutes to wash out the remaining intravascular EB. After decapitation and dissection, brain specimens were immersed into formamide (3 mL/100 mg) for 24 hours at 60°C. After centrifugation at 15 000*g* for 30 minutes at 4°C, spectrophotometric quantification of extravasated EB in the supernatants was assayed at 610 nm. EB content was quantified using a standard curve and normalized to tissue weight (μg/g).

### Neurological Deficit Score

Neurological deficits were examined by a battery of behavioral tests, as described previously,^18^ and a 28‐point neurological deficit scale was adopted. Circling behavior, climbing, front‐limb symmetry, and body symmetry were assessed. Scoring was performed by 2 trained investigators who were blinded to the animal groupings. The average score was the final score of each mouse.

### Brain Water Content

As described previously,^20^ mice brain water content was measured at days 1, 3, 5, and 7 after the ICH model was successfully constructed. Briefly, mice were anesthetized and euthanized via decapitation, and the cerebral tissues were removed. The wet weight of the brain samples was weighed immediately after euthanization. The dry weight of the brain samples was weighed after drying in an electric oven at 100°C for 24 hours. The water content was presented as a percentage of wet weight: [(wet weight−dry weight)/wet weight]×100%.

### Transfection of Short Interfering RNA in Mouse Brains

Transfection of short interfering RNA (siRNA) in mouse brains was performed as described previously.^21^ Briefly, 1 μg Mfsd2a siRNA was dissolved in 1 μL RNase‐free water. Then, 1 μL Mfsd2a siRNA, 1 μL control siRNA, and 1 μL Entranster in vivo transfection reagent were respectively diluted in 1 μL 10% glucose to obtain a final concentration of 5% glucose. Next, 2 μL Entranster in vivo was immediately added to 2 μL Mfsd2a siRNA or 2 μL control siRNA and mixed for 15 minutes at room temperature. In the ICH hemisphere, 4 μL Entranster in vivo siRNA solution was administered into striatum. Mfsd2a expression was decreased after Mfsd2a siRNA injection from day 3 to 28 following transfection (Figure S2B).

### Infection of Recombinant Adeno‐Associated Virus

The recombinant AAV‐CMV‐Mfsd2a‐ZsGreen virus (Mfsd2a overexpression) and AAV‐CMV‐ZsGreen control virus (Figure S3A) were generated by Biowit Technologies (Shenzheng, China). For in vivo infection, Mfsd2a overexpression or control virus (2 μL, 5×10^12^ viral genomes/mL) was administered intracerebroventricularly using a syringe with a 5‐gauge needle every other day until day 7.^22^, ^23^ ICH was induced 1 week after recovery from surgery. The infectivity of purified viruses was evaluated by fluorescence (510 nm) using fluorescence microscopy and Western blot, respectively.

### Western Blot

According to our previous report,^18^ protein samples from perihematomal tissues were separated by SDS‐PAGE, transferred onto polyvinylidene fluoride membranes by electroblotting, and incubated at 4°C overnight using primary antibodies against rabbit anti–mouse Mfsd2a (1:500), rabbit anti–mouse ZO‐1 (zonal occluding 1; 1:500), rabbit anti–mouse occludin (1:50 000), rabbit anti–mouse vascular endothelin (VE)–cadherin (1:1000), and rabbit anti–mouse claudin 5 (1:400), and β‐actin (1:2000) as a control. Next, the horseradish peroxidase–conjugated goat anti–rabbit secondary antibody (1:5000) was incubated with the membranes at 25°C for 1.5 hours. Chemiluminescent bands were visualized using a chemiluminescence detection system and quantified using ImageJ software.

### Immunofluorescence Staining

According to our previous methods,^24^ OCT‐embedded frozen brain tissues were cryosectioned at a thickness of 20 μm, and the sections were blocked with goat serum and then incubated with primary antibodies at 4°C overnight. After washing with PBS 3 times, the sections were incubated with second antibodies for 1 hour at room temperature. The following primary antibodies were used: goat anti–mouse Mfsd2a (1:200), rabbit anti–mouse claudin 5 (1:40), and rabbit anti–mouse CD31 (1:1000). The secondary antibodies included Alexa Fluor 647 (donkey antirabbit), and Alexa Fluor 488 (donkey antigoat).

### Transmission Electron Microscopy

Mice were anesthetized and then transcardially perfused with PBS for 1 minute, followed by 4 minutes with 5% glutaraldehyde and 4% paraformaldehyde. The brains were removed (1×1×1 mm^3^) and postfixed in the same fixative at 4°C. After fixation, the tissues were dehydrated in graded ethanol and embedded in epoxy resin. Ultrathin sections (80 nm) were then cut from the block surface, collected on copper grids, and stained with uranyl acetate and lead citrate. Brain ultrastructure was detected by a JEM‐1400Plus transmission electron microscope (JEOL, Tokyo, Japan). Twenty cortical vessels that were comparable in size from each mouse were analyzed for vesicle quantification.

### Multiple Reaction Monitoring Confirmation of Several Vesicle Trafficking–Related Proteins

This experiment was performed as previously described^25^ with some modifications in Triple Quad 6500 liquid chromatography–tandem mass spectrometry (AB SCIEX, Concord, Canada). Briefly, a C18 analytical column was prepared (0.075×150 mm, 3 μm, 120 A), and 2 mg of brain peptides was subjected to the EMS‐EPI (Enhanced Mass Spectrum‐Enhanced Product Ion) mode for identification. Then, the EMS‐EPI data were searched with ProteinPilot (AB SCIEX). Transitions for all identified peptides were designed by importing the ProteinPilot results files to MRMPilot (Skyline software; AB SCIEX) with the peptide selection criteria including a unique peptide for a protein and no missed cleavage. Finally, the 67 designed peptides (3 proteins with 5 peptides, 3 proteins with 4 peptides, 5 proteins with 3 peptides, 5 proteins with 2 peptides, and 15 proteins with 1 peptide) and 378 transitions were used to survey the protein digests from each brain using the multiple reaction monitoring (MRM) mode with 3 injection repeats. For MRM confirmation, the raw MRM data were processed using Skyline 3.5.9.10130.

### Statistical Analysis

All data are expressed as mean±SD. The significance of differences between 2 and ≥3 groups was determined using nonparametric tests, the Student *t* test, or 1‐way ANOVA followed by the Scheffe post hoc test. Two‐way ANOVA with repeated measures was performed if appropriate to compare repeated measures data, and the main effects of the genotype (treatment) and time points and the interaction were assessed. Statistical differences were considered significant if the *P* value was <0.05.

## Results

### Mfsd2a Protein Expression Decreases After ICH

**Figure 1:**
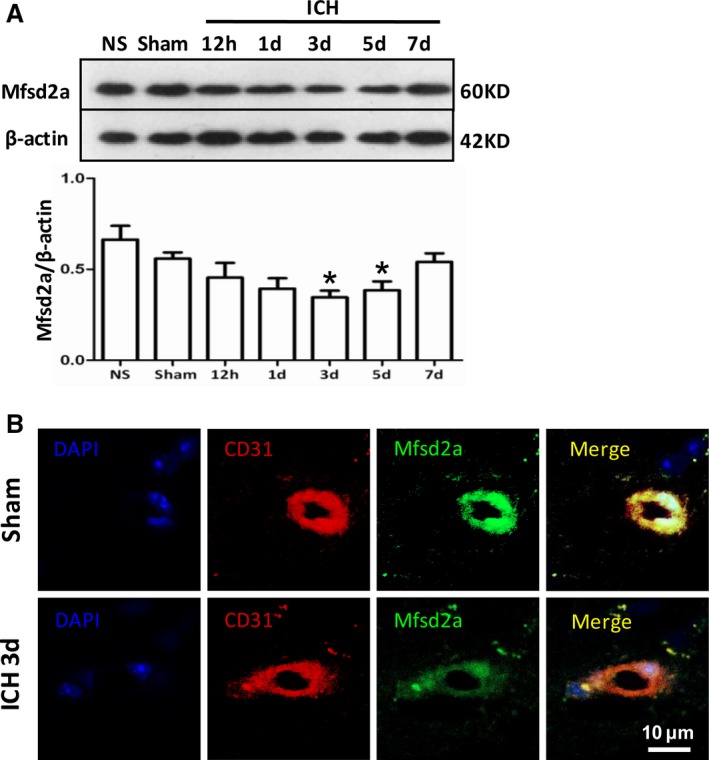
Mfsd2a (major facilitator superfamily domain containing 2a) reductions in brain capillary following intracerebral hemorrhage (ICH). A, Mfsd2a protein expression at different time points after intracerebral hemorrhage using Western blot. B, Immunostaining for Mfsd2a (green), CD31‐positive endothelial capillary profiles (red) in brain microvessels from sham and ICH mice at 3 days. The merged images of the overlay of Mfsd2a together with CD31 are shown as yellow, and the nuclei were stained with DAPI (4′,6‐diamidino‐2‐phenylindole; blue). Scale bar=10 μm. *P<0.05 compared with sham group.

To investigate the role of Mfsd2a in BBB injury after ICH, we first evaluated the protein expression pattern of Mfsd2a in perihematomal brain tissues after ICH by Western blot and immunofluorescent staining. The Western blot results showed that Mfsd2a protein levels in the perihematomal tissues were significantly decreased in the ICH mice compared with the sham group at day 3 (34.63±3.63% versus 55.87±3.44%, *P*=0.0355) and day 5 (38.50±4.89% versus 55.87±3.44%, *P*=0.0401) after ICH (Figure 1A). Mfsd2a is selectively expressed in BBB‐containing blood vessels; therefore, we measured the expression of Mfsd2a on CD31‐positive endothelial cells after ICH by immunofluorescence. We found that Mfsd2a levels in cerebral microvessels were decreased in CD31‐positive endothelial cells in the ICH mice compared with sham mice (Figure 1B). These findings suggest that decreased Mfsd2a expression in the brain microvessels surrounding the perihematomal tissues may be involved in BBB injury after ICH.

### Downregulation of Mfsd2a by siRNA Increases BBB Permeability and Aggravated Neurological Dysfunction After ICH

**Figure 2:**
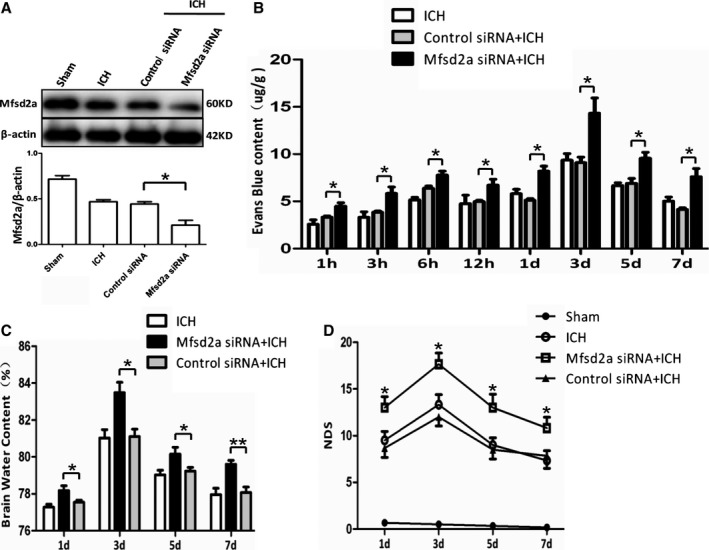
Knockdown of Mfsd2a (major facilitator superfamily domain containing 2a) by short interfering RNA (siRNA) increased blood–brain barrier (BBB) permeability and accelerated neurological dysfunction with intracerebral hemorrhage (ICH). A, Mice were pretreated with a single intracerebroventricular injection of 1 μg Mfsd2a siRNA or control siRNA. The expression of Mfsd2a was detected by immunoblots after ICH at day 3. *P<0.05 vs ICH, n=6. B, BBB permeability increased at different time points after ICH in the Mafsd2a siRNA+ICH group. *P<0.05 vs control siRNA+ICH group, n=6. C, Brain water content and (D) The neurological deficit score (NDS) of sham, ICH, Mafsd2a siRNA+ICH, and control siRNA+ICH groups at 1, 3, 5, and 7 days after the onset of ICH. *P<0.05, **P<0.01 vs the control siRNA+ICH groups at the corresponding time points, n=6. Two‐way ANOVA showed a significant difference in main effects of all treatment groups (*P*<0.05) but not of time points (*P*>0.05), and there was no interaction between treatments and time points (*P*>0.05).

To further study the role of Mfsd2a in ICH‐induced BBB injury, we injected Mfsd2a siRNA to knockdown Mfsd2a in the brain; this resulted in decreased Mfsd2a at 3 days after transfection (Figure S2B). We verified that Mfsd2a protein expression was significantly decreased in the Mfsd2a siRNA group compared with the control siRNA group after ICH (21.04±5.38% versus 46.81±2.18%, *P*=0.0194) (Figure 2A). Next, we investigated the effects of Mfsd2a knockdown on BBB injury after ICH. BBB permeability was measured using the EB technique (Figure S2C). The Mfsd2a siRNA group showed increased extravasation of EB in the ipsilateral hemisphere at different time points compared with the control siRNA group after ICH (Figure 2B). Moreover, the brain water content and the neurological deficit score in the Mfsd2a siRNA group were significantly higher compared with the control siRNA group after ICH (Figure 2C and 2D). Taken together, our results indicate that downregulation of Mfsd2a increases BBB permeability and aggravates neurological dysfunction in a mouse model of ICH.

### Mfsd2a^−/−^ Mice Exhibited Significantly Increased BBB Injury and Neurological Deficits Caused by ICH

**Figure 3:**
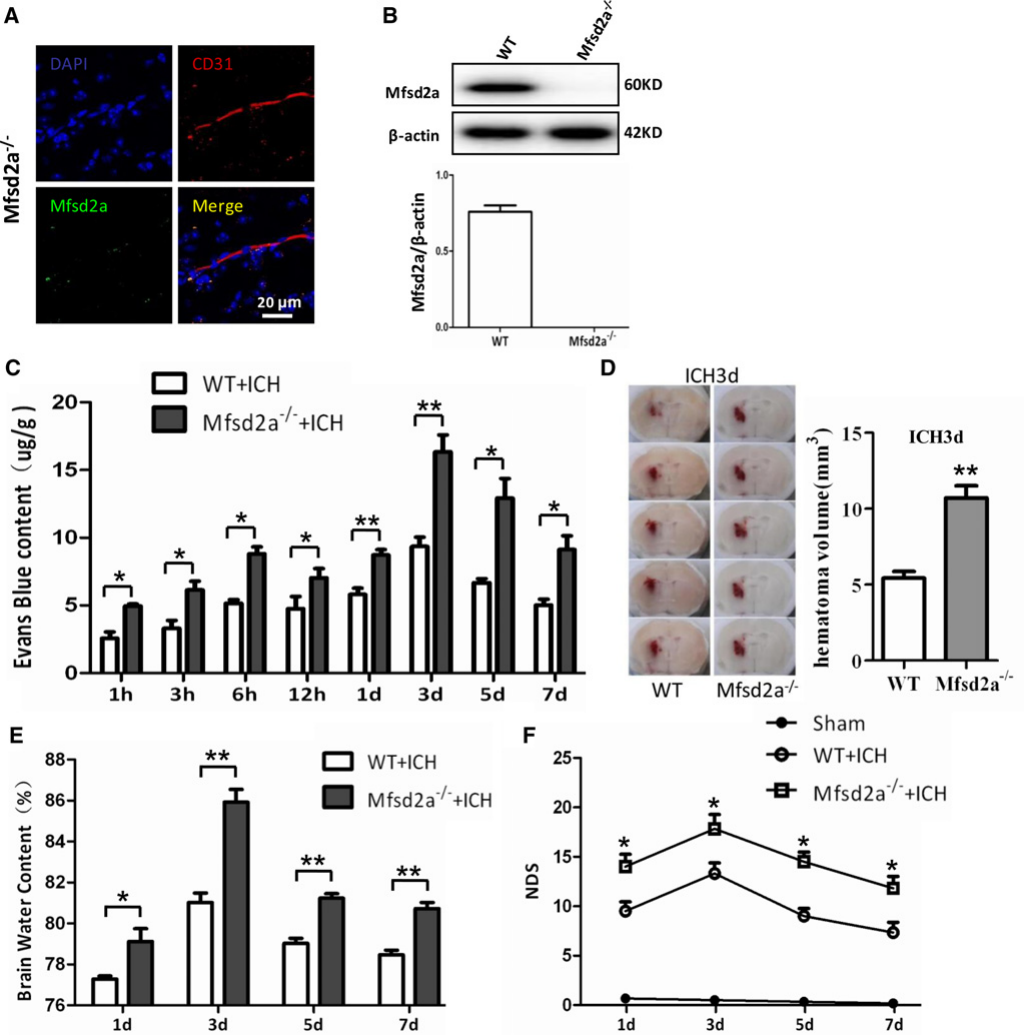
Increased blood–brain barrier (BBB) permeability and enhanced neurological dysfunction in Mfsd2a (major facilitator superfamily domain containing 2a) knockout (Mfsd2a^−/−^) mice with intracerebral hemorrhage (ICH). A, Immunostaining for Mfsd2a (green) and CD31‐positive endothelial capillary profiles (red) in brain microvessels from Mfsd2a knockout (Mfsd2a^−/−^) mice. Scale bar=20 μm. B, Detection of Mfsd2a expression using Western blot. C, BBB permeability increased at different time points after ICH in Mfsd2a^−/−^+ICH mice. *P<0.05, **P<0.01 vs wild type (WT) plus ICH mice, n=6. D, Representative coronal sections from WT+ICH and Mfsd2a^−/−^+ICH mice. The hematoma volume was calculated. **P<0.01 vs WT+ICH mice, n=6. E, Brain water content of WT+ICH and Mfsd2a^−/−^+ICH mice. *P<0.05, **P<0.01 vs WT+ICH mice, n=6. F, The neurological deficit score (NDS) of sham, WT+ICH, and Mfsd2a^−/−^+ICH mice at 1, 3, 5, and 7 days after the onset of ICH. *P<0.05 vs WT+ICH mice at the corresponding time points, n=6. Two‐way ANOVA showed a significant difference in main effects of all treatment groups (*P*<0.05) but not of time points (*P*>0.05), and there was no interaction between treatments and time points (*P*>0.05).

To further evaluate the Mfsd2a function in BBB injury after ICH, we constructed an Mfsd2a^−/−^ mouse that was verified by immunofluorescent staining (Figure 3A) and Western blot (Figure 3B). Mfsd2a expression was not present in Mfsd2a^−/−^ mice. The EB content showed that the BBB permeability in Mfsd2a^−/−^ mice was significantly increased (Figure S2C and S2D) compared with wild‐type (WT) mice at 1 hour (4.75±0.35 versus 2.57±0.46 μg/g, *P*=0.0198), 3 hours (6.14±0.65 versus 3.31±0.56 μg/g, *P*=0.031), 6 hours (8.57±0.72 versus 5.15±0.27 μg/g, *P*=0.0115), 12 hours (7.37±0.37 versus 4.71±0.87 μg/g, *P*=0.0495), 1 day (8.72±0.41 versus 5.81±0.46 μg/g, *P*=0.0093), 3 days (16.35±1.25 versus 9.36±0.68 μg/g, *P*=0.0081), 5 days (12.93±1.45 versus 6.67±0.28 μg/g, *P*=0.0135), and 7 days (9.11±1.03 versus 5.02±0.44 μg/g, *P*=0.0219; Figure 3C) after ICH. In this experiment, hematoma volume in the Mfsd2a^−/−^ mice were increased compared with those in the WT mice after ICH (10.70±0.81 versus 5.42±0.44 μL, *P*=0.0013; Figure 3D). Meanwhile, we also found that the brain water content (Figure 3E) and the neurological deficit score (Figure 3F) in Mfsd2a^−/−^ mice were significantly higher than those in the WT mice at the same time points after ICH. These results suggest that Mfsd2a^−/−^ significantly increases BBB permeability and neurological dysfunction in ICH mice.

### Overexpression of Mfsd2a Decreases BBB Permeability and Neurological Dysfunction With ICH

**Figure 4:**
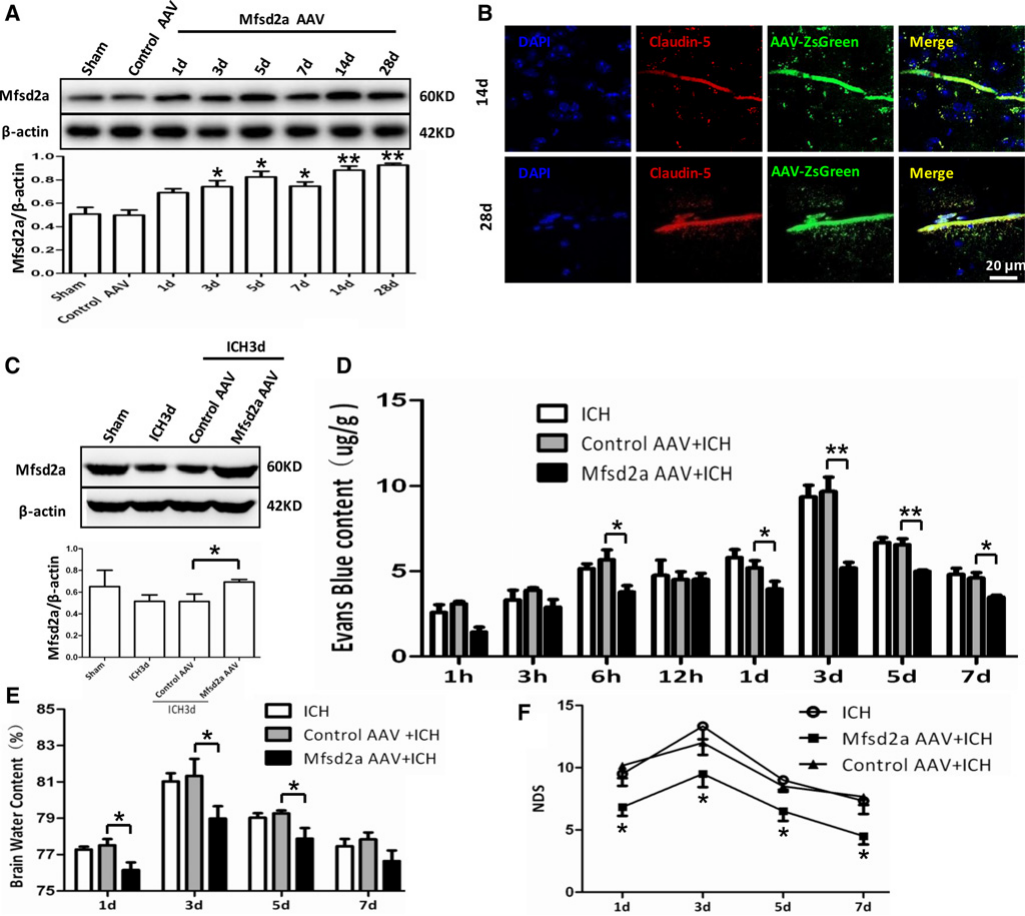
Enhanced Mfsd2a (major facilitator superfamily domain containing 2a) expression after injection with Mfsd2a overexpression virus in perihematomas following intracerebral hemorrhage (ICH). A, Mfsd2a protein expression at different time points after injection with Mfsd2a overexpression virus (Mfsd2a adeno‐associated virus [AAV]) using Western blot. *P<0.05, **P<0.01 vs control AAV group, n=6. B, Immunostaining for ZsGreen (green) and claudin 5–positive endothelial capillary profiles (red) in brain microvessels. The merged images of the overlay of ZsGreen together with claudin 5 are shown as yellow, and the nuclei are stained with DAPI (4′,6‐diamidino‐2‐phenylindole; blue). Scale bar=20 μm. C, Detection of Mfsd2a expression in mice injected with Mfsd2a overexpression virus and subjected to ICH, using Western blot. *P<0.05 vs control AAV+ICH group, n=6. D, Blood–brain barrier (BBB) permeability decreased at different time points after ICH in the Mfsd2a AAV+ICH group. *P<0.05, **P<0.01 vs control AAV+ICH group, n=6. E, Brain water content of ICH, control AAV+ICH, and Mfsd2a AAV+ICH groups. *P<0.05 vs control AAV+ICH, n=6. F, The neurological deficit score (NDS) of ICH, control AAV+ICH, and Mfsd2a AAV+ICH groups at 1, 3, 5, and 7 days after the onset of ICH. *P<0.05 vs the control AAV+ICH group at the corresponding time points, n=6. Two‐way ANOVA showed a significant difference in main effects of all treatment groups (*P*<0.05) but not of time points (*P*>0.05), and there was no interaction between treatments and time points (*P*>0.05).

We showed that knockdown or knockout of Mfsd2a aggravated BBB injury and neurological dysfunction after ICH. Consequently, we investigated whether overexpression of Mfsd2a could reduce BBB injury and improve neurological deficits caused by ICH, as well as reverse the effects of knockdown and knockout. We injected the AAV‐CMV‐Mfsd2a virus to overexpress Mfsd2a in brains and investigated the effects of Mfsd2a upregulation on BBB and neurological functions (Figure S3B). We found that Mfsd2a protein expression gradually increased at day 3 and steadily increased at day 14 and even day 28 after injection, detected by both Western blot (Figure 4A) and immunofluorescent staining (Figure 4B, S3C, and S3D) compared with the control virus group. Next, we measured Mfsd2a protein levels after ICH and found that Mfsd2a protein levels were significantly increased following Mfsd2a overexpression (Mfsd2a adeno‐associated virus [AAV]) after ICH compared with control AAV (Figure 4C). Next, we examined BBB permeability using EB and showed that the Mfsd2a AAV group exhibited decreased extravasation of EB in the ipsilateral hemisphere compared with the control AAV group at 6 hours (3.78±0.37 versus 5.15±0.27 μg, *P*=0.0409), 1 day (3.92±0.45 versus 5.81±0.46 μg, *P*=0.0443), 3 days (5.19±0.32 versus 9.36±0.67 μg, *P*=0.0051), 5 days (4.99±0.07 versus 6.67±0.28 μg, *P*=0.0048), and 7 days (3.48±0.10 versus 4.82±0.35 μg, *P*=0.0233) after ICH (Figure 4D). The brain water content in the Mfsd2a AAV group was also significantly lower compared with the control AAV group at 1 day (76.15±0.42% versus 77.28±0.15%, *P*=0.0447), 3 days (78.79±0.70% versus 81.03±0.45%, *P*=0.0369), and 5 days (77.63±0.49% versus 79.03±0.24%, *P*=0.0442) after ICH (Figure 4E). Moreover, the Mfsd2a AAV group showed significant improvement in neurological deficits compared with controls, as shown by the lower neurological deficit score in the Mfsd2a AAV group compared with the control group at 1 day (6.83±0.70 versus 9.50±0.95, *P*=0.0395), 3 days (9.66±0.95 versus 13.33±1.05, *P*=0.0403), 5 days (6.50±0.76 versus 9.00±0.77, *P*=0.0477), and 7 days (4.50±0.67 versus 7.33±1.05, *P*=0.0411) after ICH (Figure 4F). Together, these results support the concept that that overexpression of brain endothelial Mfsd2a could attenuate ICH‐induced BBB disruption.

### The Protective Effects of Mfsd2a on BBB Injury May Be Involved in the Inhibition of Vesicular Transcytosis of Cerebral Vascular Endothelial Cells After ICH

**Figure 5:**
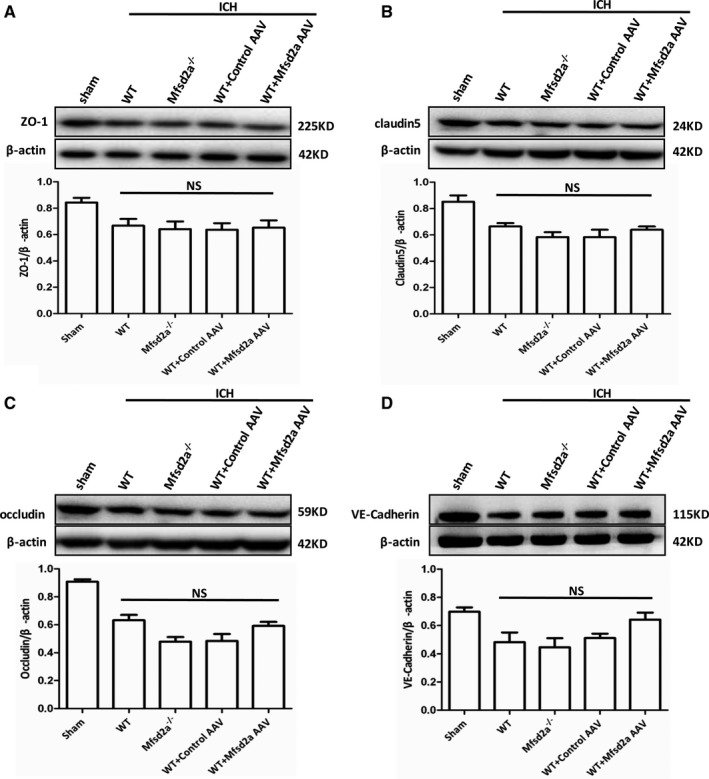
Protein levels of tight junction–associated proteins in intracerebral hemorrhage microvessels were detected by Western blot after intracerebral hemorrhage (ICH). Representative protein expressing bands of ZO‐1 (zonal occluding 1) (A), claudin 5 (B), occludin (C), and vascular endothelin (VE)–cadherin (D) in sham, wild type (WT) plus ICH, Mfsd2a (major facilitator superfamily domain containing 2a) knockout (Mfsd2a^−/−^) plus ICH, as well as control adeno‐associated virus (AAV) plus ICH and Mfsd2a AAV+ICH groups. n=6. NS indicates not significant.

We showed that Mfsd2a is selectively expressed in cerebral vascular endothelial cells and plays an important role in reducing BBB injury after ICH; however, the mechanisms of Mfsd2a on protecting BBB are still unknown. We first measured the tight junction proteins, which were demonstrated to be involved in BBB regulation, and found that the expressions of ZO‐1 (Figure 5A), claudin 5 (Figure 5B), occludin (Figure 5C), and VE‐cadherin (Figure 5D) in the perihematomal brain tissues were all decreased after ICH. Manipulation of Mfsd2a, however, showed no differences in these tight junction protein levels among the WT, Mfsd2a^−/−^, the control AAV and Mfsd2a AAV mice after ICH. These data suggested that the protective roles of Mfsd2a in BBB injury are not related to damage of tight junction after ICH.

**Figure 6:**
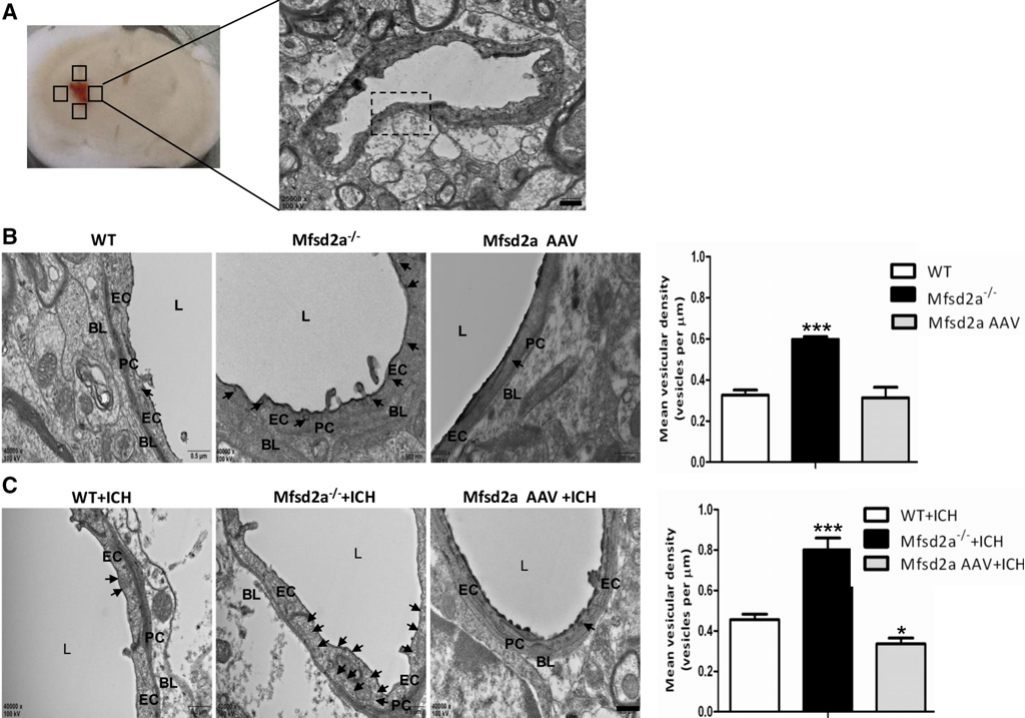
Ultrastructural observation (×40 000) of mice. A, Coronal sections show collection of cerebral tissues from the perihematomal region and the selection of fields of view for ultrastructural observation (scale bar=1 μm). B, Electron microscopy revealed more vesicles (arrows) of endothelial cells (ECs) in Mfsd2a (major facilitator superfamily domain containing 2a) knockout (Mfsd2a^−/−^) mice compared with wild‐type (WT) mice. Vesicular density quantification and the number of EC vesicles in Mfsd2a knockout (Mfsd2a^−/−^) mice increased compared with WT mice (20 vessels of similar size [4‐ to 5‐μm lumen] per mouse were analyzed, n=3). ***P<0.001 vs WT mice. C, The EC vesicles (arrows) were revealed in WT plus intracerebral hemorrhage (ICH) and Mfsd2a^−/−^+ICH mice, respectively. Vesicular density quantification showed that the number of EC vesicles in Mfsd2a^−/−^+ICH mice was increased compared with WT+ICH mice (20 vessels of similar size [4‐ to 5‐μm lumen] per mouse were analyzed, n=3). The number of EC vesicles in the Mfsd2a adeno‐associated virus (AAV) plus ICH group was decreased compared with the control AAV+ICH group. *P<0.05, ***P<0.001 vs control AAV+ICH group. Scale bar=500 nm. BL indicates basal lamina; L, lumen; PC, pericyte.

Next, we used electron microscopy (EM) to further observe the changes in micromorphology of cerebral vessels after ICH. The ultrastructure of the BBB was observed in the perihematomal brain tissues from coronal sections of the brain (Figure 6A). The results showed that the EM ultrastructure of endothelial tight junctions was normal and consistent among WT, Mfsd2a^−/−^, the control AAV and Mfsd2a AAV mice after ICH (Figure 6B). Tight junctions in these mice appeared similarly with electron‐dense linear structures in which adjacent membranes were lined up tightly (Figure 6B). However, EM analysis showed increased caveolae‐like vesicles that are partly responsible for peripheral endothelial permeability in Mfsd2a^−/−^ mice compared with WT mice (Figure 6B and 6C). After ICH, we revealed increased complexity of the vesicles in Mfsd2a^−/−^ mice compared with WT mice. Meanwhile, more vesicles were revealed in Mfsd2a^−/−^ mice (0.75±0.04 versus 0.45±0.02, *P*=0.0007), whereas fewer vesicles (0.34±0.03 versus 0.45±0.02, *P*=0.0417) were shown in Mfsd2a AAV compared with WT mice after ICH. Together, these findings suggest that the BBB leakiness observed in Mfsd2a^−/−^ ICH mice may be caused not by opening of tight junctions but rather by increased transcellular trafficking across the endothelial cytoplasm.

**Figure 7:**
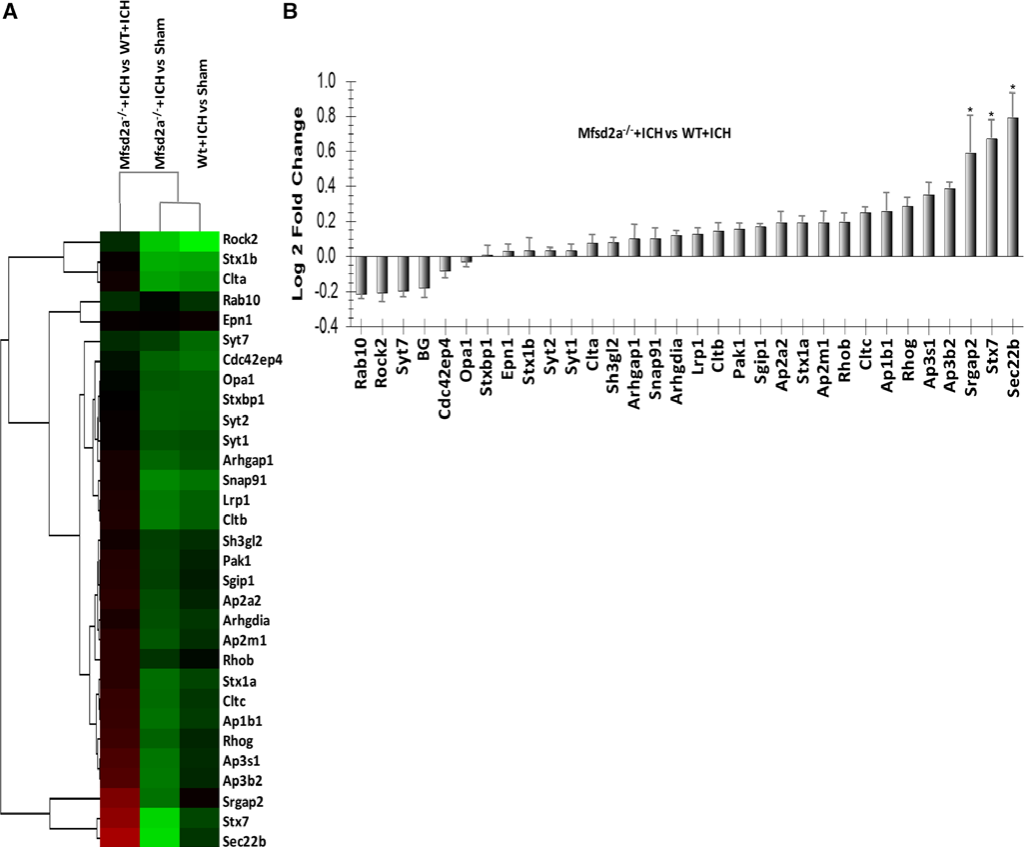
Verification of vesicle trafficking–related proteins in mouse samples using multiple reaction monitoring (MRM). A, Cluster map was displayed to compare the protein expression patterns of wild type (WT) plus intracerebral hemorrhage (ICH), Mfsd2a (major facilitator superfamily domain containing 2a) knockout (Mfsd2a^−/−^) plus ICH, and sham mice. Red indicates higher expression, green indicates lower expression, and black indicates the same expression levels in the 2 strains. B, List of relative abundance of differentially expressed vesicle trafficking–related proteins identified in Mfsd2a^−/−^+ICH and WT+ICH samples. Several vesicle trafficking–related proteins in Mfsd2a^−/−^+ICH were significantly higher than those in WT+ICH. The log_2_ (Ratio) were calculated by method of pair‐ wise combination and error weighted average. Then the significant differentially expressed genes were filtered by the criteria of log_2_ (Ratio) > = 0.585 and *P‐value < 0.05.

Previous studies have shown that vesicle trafficking–related proteins were related to BBB permeability regulation^26^, ^27^; therefore, we investigated whether Mfsd2a influenced these vesicle trafficking–related proteins to regulate vesicular transcytosis after ICH. In total, 31 vesicle trafficking–related proteins were detected in perihematomal brain tissues of WT, Mfsd2a^−/−^, and sham mice in ICH models using MRM. A cluster analysis of the proteins identified in the WT+ICH, Mfsd2a^−/−^+ICH, and sham mice was conducted using Skyline 3.5.9.10130, and the proteins were grouped according to their expression levels (Figure 7A). The 31 vesicle trafficking–related proteins included 26 that were upregulated and 5 that were downregulated (Mfsd2a^−/−^+ICH versus WT+ICH; Figure 7B). Compared with the WT+ICH mice, 3 proteins (3 upregulated and 0 downregulated) including Srgap2 (Slit‐Robo Rho GTPase activating protein 2), Stx7 (syntaxin 7), and Sec22b (SEC22 homolog B, vesicle trafficking protein) showed significant changes in abundance in the Mfsd2a^−/−^+ICH mice, suggesting that these mice were more sensitive to BBB permeability and might be candidates for early indicators of vesicle trafficking in Mfsd2a^−/−^ mice.

## Discussion

A key pathological manifestation of post‐ICH brain injury is BBB disruption, which contributes to further parenchymal damage and edema.^28^ Consequently, protecting the BBB seems pressingly necessary and is considered a promising disease‐control strategy for the treatment of brain injury in the early period after ICH.^6^ Our study is the first to show that Mfsd2a has a protective role in ICH‐induced BBB disruption. We demonstrated that Mfsd2a expression was significantly decreased in perihematomal tissues in mice with ICH. Enhanced expression of brain endothelial Mfsd2a attenuated ICH‐induced BBB disruption. In addition, Mfsd2a regulated the BBB function via regulating vesicular transcytosis across the cerebral endothelium.

Significant progress has been made on the causes of BBB dysfunction after ICH. Many pathogenic factors, such as blood components (eg, thrombin, hemoglobin, iron) and inflammation,^3^, ^6^ contribute to BBB dysfunction. It should be noted, however, that blood components or inflammation of pathogenic factors in ICH‐induced BBB injury may similarly activate downstream pathways that enhance BBB permeability. BBB dysfunction, exhibited mostly as increased permeability, is an important molecular and cellular event that contributes to aggravating brain injury after ICH.^1^ Between the peripheral circulation and the central nervous system, the BBB provides a selective barrier‐limiting permeability and thus defines a stable environment crucial for brain homeostasis,^29^, ^30^ which is a unique brain endothelial physiological barrier sealing the central nervous system and controlling substance influx and efflux.^9^ Under normal conditions, the paracellular pathway (alterations in tight junction function) and/or the transcellular route (vesicular transcytosis) modulates BBB permeability via the brain microvascular endothelial cells.^10^ The tightness of those junctions and a relatively low level of vesicular transcytosis result in low BBB permeability.^9^

Many tight junction–related proteins have been identified to control BBB permeability through modulation of the structural components of the tight junction. Previous reports have demonstrated that ZO‐1, claudin 5, occludin, and VE‐cadherin can be considered sensitive indicators of the BBB in both normal and disturbed functional states.^11^, ^31^, ^32^ Our findings revealed that Mfsd2a protein expression was decreased in the ICH model. In addition, mice with Mfsd2a loss‐of‐function or global deficiency developed significantly enhanced permeability of BBB and had higher neurological deficit score test performance, whereas overexpression of brain endothelial Mfsd2a attenuated ICH‐induced BBB disruption. However, reduction and overexpression of Mfsd2a in brains subjected to ICH resulted in no significant changes in tight junction–related proteins including ZO‐1, claudin 5, occludin, and VE‐cadherin. The results provide evidence that the protective roles of Mfsd2a in BBB may not be related to damage of the tight junction after ICH.

We further investigated the brain vessel morphology in Mfsd2a deficiency and overexpression by EM. The EM analysis showed a significant increase in the quantity of pinocytotic vesicles in brain vessel endothelium of Mfsd2a^−/−^ mice, and this is consistent with the previous findings.^16^ In our study, we specifically aimed to interfere with the BBB breakdown in mouse models of ICH.

More pinocytotic vesicles were observed in Mfsd2a^−/−^ mice than in mice with Mfsd2a overexpression after ICH. Our results suggest that Mfsd2a plays an important role in regulating permeability of the BBB via the transcellular transport pathway (vesicular transcytosis). Previous studies focused mostly on paracellular permeability of the BBB, and the paracellular pathway is subject to extensive research.^33^, ^34^ Moreover, the low prevalence of nonspecific transcytotic events is another important feature of the BBB endothelium including (macro)pinocytosis and the subsequent vesicles trafficking to the opposite membrane.^12^ Although less studied, there are many examples for a transcellular route across the BBB in pathological conditions in which increased BBB permeability does not correlate with the alteration in tight junction organization.^35^, ^36^ Small and tortuous channels that meander through discontinuities in the intercellular junctional complex form the paracellular transport route. Diffusion of molecules through the paracellular transport route is size‐limited,^37^ whereas an energy‐requiring receptor can mediate vesicular transcytosis to transport macromolecules. In fact, vesicular transcytosis appears to be the most promising mechanism for improvement of drug delivery through the brain barrier.^38^

To more directly assess whether a link exists between Mfsd2a and vesicular transcytosis, we investigated whether Mfsd2s influenced these vesicle trafficking–related proteins to regulate vesicular transcytosis after ICH. We used a targeted quantification method (MRM) to verify several potential vesicle trafficking–related proteins in WT+ICH and Mfsd2a^−/−^+ICH mice. Compared with WT+ICH mice, it is worth noting that 3 of the significantly increased proteins (Srgap2, Stx7, and Sec22b) were found in Mfsd2a^−/−^+ICH mice, and the finding may provide clues to further pursue the molecular events of vesicle‐mediated transcytosis in the early stage of ICH. Previous studies have shown that Stx7, as the endosomal SNARE protein, could regulate vesicle‐trafficking events involved in phagocytosis and cytokine secretion via transcytosis.^39^, ^40^, ^41^ Srgap2, as one of the srGAP family of Rho GAP proteins, has been mainly shown to regulate membrane protrusion, cell spreading, and migration.^42^, ^43^, ^44^ Sec22b is a SNARE and SNARE‐associated protein and functions in the trafficking between the endoplasmic reticulum and the Golgi via a conserved nonfusogenic mechanism in plasma membrane expansion.^45^ Sec22b‐mediated SNARE bridges could provide a direct balance between nonvesicular and vesicular transport.^46^ Accordingly, based on our results showing that Srgap2, Stx7, and Sec22b were significantly changed in Mfsd2a^−/−^ mice after ICH and that the role of Mfsd2a may be related to the transcytosis, we speculate that Stx7 may be involved in transcytosis after ICH. Nevertheless, the exact roles of the 3 molecules (Srgap2, Stx7, and Sec22b) and whether Stx7 is related to transcytosis after ICH should be further investigated.

Our study lacks results from human ICH samples, and this may limit the applicability of the findings to ICH in humans; therefore, we should further investigate in human samples from ICH. In conclusion, our findings suggest that Mfsd2a regulates permeability of the BBB via vesicular transcytosis, not the tight junction, and thus could be a potential therapeutic target for ICH drug delivery.

## Sources of Funding

This work was supported by the National Basic Research Program of China (973 Program) (2014CB541605), National Science Foundation of China (81601028), and the National Natural Science Fund for Distinguished Young Scholars (81525008).

## Disclosures

None.

## Supporting information


**Figure S1.** Generation of Mfsd2a (major facilitator superfamily domain containing 2a) knockout (Mfsd2a^−/−^) mice. A, Mouse EGE‐WJL‐003 gene spans about 14.34 kb on the chromosome 4 reverse strand. Gene ID:76574. B, Schematic strategy for generation of Mfsd2a^−/−^ mice via the EGE targeting strategy.
**Figure S2.** Reduction of Mfsd2a (major facilitator superfamily domain containing 2a) increased the permeability of the blood–brain barrier (BBB). A, Coronal sections show collection of cerebral tissues from the perihematomal region. B, Changes of Mfsd2a expression at different time points after injection with Mfsd2a short interfering RNA (siRNA) using Western blot. *P<0.05 vs sham mice, n=6. C, The photograph shows mouse brains that received an intravenous infusion of Evans blue dye (EB). In normal mice, the BBB was intact and EB extravasation was absent. The bluish colors of the brain's hemispheres show the occurrence of BBB disruption in intracerebral hemorrhage (ICH), Mfsd2a siRNA+ICH, and Mfsd2a knockout (Mfsd2a^−/−^) plus ICH mice. D, After EB injection, red EB fluorescence (emission: 680 nm) was enhanced in Mfsd2a^−/−^ slices (white arrows).
**Figure S3.** Enhanced Mfsd2a (major facilitator superfamily domain containing 2a) expression injected with Mfsd2a overexpression virus in perihematomas. A, Schematic diagram of pAAV‐Mfsd2a plasmid. Mfsd2a fragments were amplified by polymerase chain reaction and cloned into plasmid AAV (pAAV)‐IRES‐ZsGreen vector by BamHI, EcoRI, Xbal, Xhol, Agel, Mfel, and Spel to construct the pAAV‐Mfsd2a plasmid. B, At 2 weeks after injection of Mfsd2a overexpression virus, significant expression of AAV‐ZsGreen was noted. C, Immunostaining for control‐ZsGreen (green) and claudin 5–positive endothelial capillary profiles (red) in brain microvessels. The merged images of the overlay of ZsGreen together with claudin 5 are shown as yellow, and the nuclei are stained with DAPI (4′,6‐diamidino‐2‐phenylindole; blue). Scale bar=20 μm. D, Immunostaining for AAV‐ZsGreen (green) and CD31‐positive endothelial capillary profiles (red) in brain microvessels from mice injected with Mfsd2a overexpression virus. The merged images of the overlay of AAV‐ZsGreen together with claudin 5 are shown as yellow, and the nuclei were stained with DAPI (blue). Scale bar=10 μm.
