# Supplementary material for: Mfsd2a (Major Facilitator Superfamily Domain Containing 2a) Attenuates Intracerebral Hemorrhage–Induced Blood–Brain Barrier Disruption by Inhibiting Vesicular Transcytosis
Source: J Am Heart Assoc. 2017 Jul 19;6(7):e005811. doi: 10.1161/JAHA.117.005811 (PMC5586300; doi:10.1161/JAHA.117.005811)
Supplement: Supplementary file 1 — Figure S1. Generation of Mfsd2a (major facilitator superfamily domain containing 2a) knockout (Mfsd2a−/−) mice. A, Mouse EGE‐WJL‐003 gene spans about 14.34 kb on the chromosome 4 reverse strand. Gene ID:76574. B, Schematic strategy for generation of Mfsd2a−/− mice via the EGE targeting strategy. Figure S2. Reduction of Mfsd2a (major facilitator superfamily domain containing 2a) increased the permeability of the blood–brain barrier (BBB). A, Coronal sections show collection of cerebral tissues from the perihematomal region. B, Changes of Mfsd2a expression at different time points after injection with Mfsd2a short interfering RNA (siRNA) using Western blot. *P<0.05 vs sham mice, n=6. C, The photograph shows mouse brains that received an intravenous infusion of Evans blue dye (EB). In normal mice, the BBB was intact and EB extravasation was absent. The bluish colors of the brain's hemispheres show the occurrence of BBB disruption in intracerebral hemorrhage (ICH), Mfsd2a siRNA+ICH, and Mfsd2a knockout (Mfsd2a−/−) plus ICH mice. D, After EB injection, red EB fluorescence (emission: 680 nm) was enhanced in Mfsd2a−/− slices (white arrows). Figure S3. Enhanced Mfsd2a (major facilitator superfamily domain containing 2a) expression injected with Mfsd2a overexpression virus in perihematomas. A, Schematic diagram of pAAV‐Mfsd2a plasmid. Mfsd2a fragments were amplified by polymerase chain reaction and cloned into plasmid AAV (pAAV)‐IRES‐ZsGreen vector by BamHI, EcoRI, Xbal, Xhol, Agel, Mfel, and Spel to construct the pAAV‐Mfsd2a plasmid. B, At 2 weeks after injection of Mfsd2a overexpression virus, significant expression of AAV‐ZsGreen was noted. C, Immunostaining for control‐ZsGreen (green) and claudin 5–positive endothelial capillary profiles (red) in brain microvessels. The merged images of the overlay of ZsGreen together with claudin 5 are shown as yellow, and the nuclei are stained with DAPI (4′,6‐diamidino‐2‐phenylindole; blue). Scale bar=20 μm. D, Immunostaining for AAV‐ [file JAH3-6-e005811-s001.pdf]

Supplemental Material

Figure S1. Generation of Mfsd2a knockout mice.

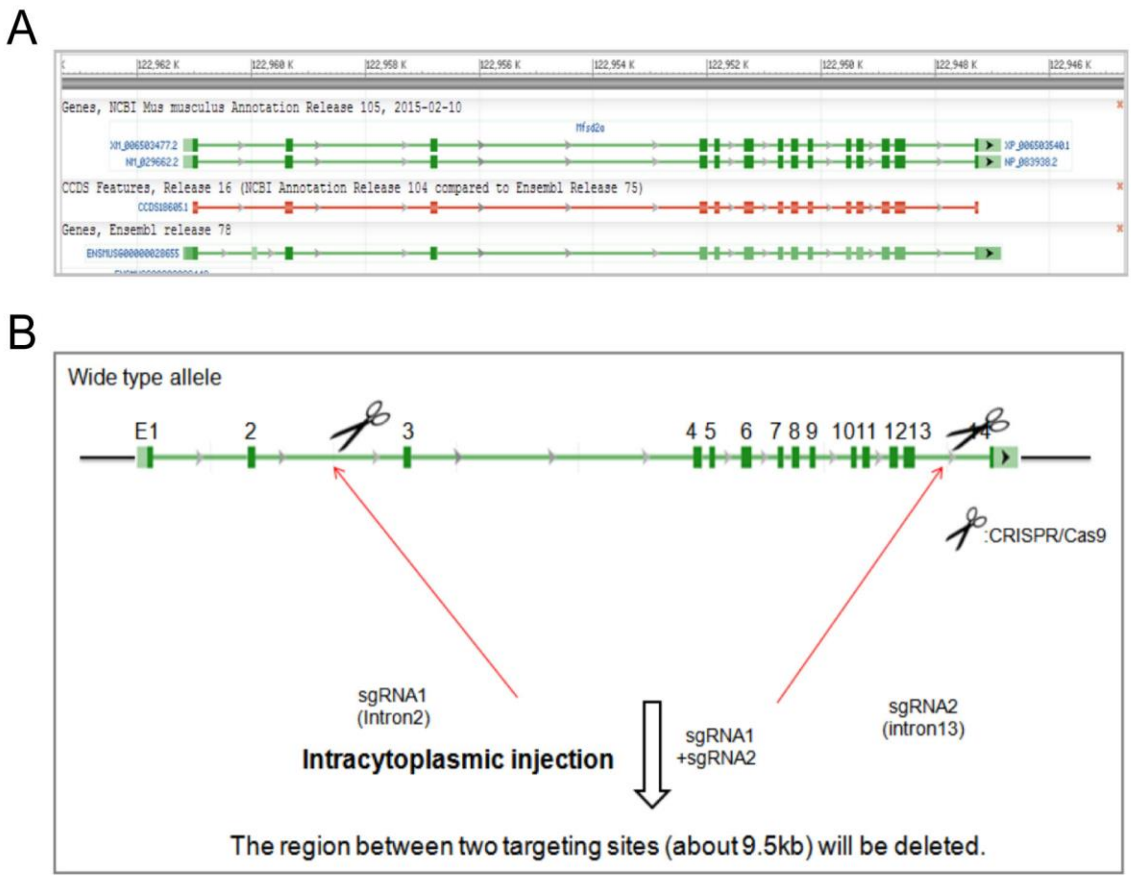

(A)Mouse EGE-WJL-003 gene spans about 14.34kb on chromosome 4 reverse strand. Gene ID:76574. (B)Schematic strategy for generation of Mfsd2a knockout mice via EGE Targeting strategy.

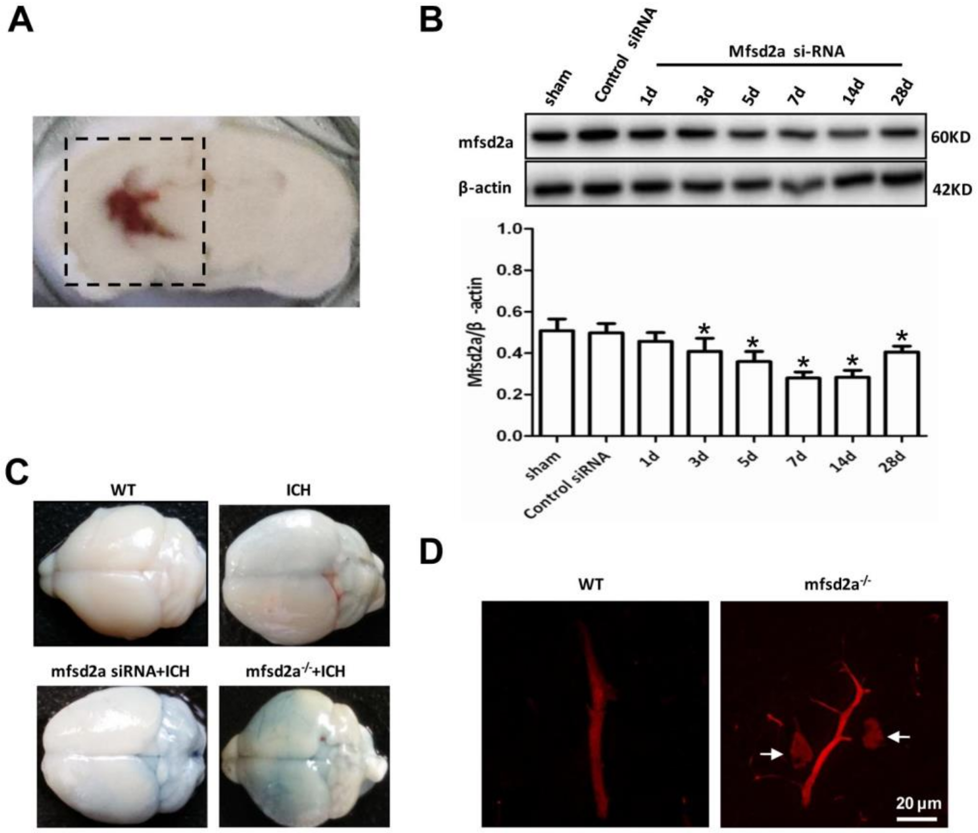

Figure S2. Reductin of Mfsd2a increased the permeability of BBB.

(A) Coronal sections show collection of cerebral tissues from the perihematomal region. (B)Changes of Mfsd2a expression at different time points after injection with Mfsd2a-siRNA using Western blot. *P<0.05 versus Sham mice, n = 6. (C) The photograph is showing the mice brains that received an IV infusion of Evans blue (EB) dye. In normal mice the blood–brain barrier (BBB) was intact and the EB extravasation was absent. The bluish colors of the brain's hemispheres show the occurrence of BBB disruption in ICH, Mfsd2a siRNA+ICH,and Mfsd2a$^{-/-}$ +ICH mice. (D)After Evans blue injection,red Evans blue fluorescence (Em: 680 nm) is enhanced in Mfsd2a$^{-/-}$ slices(white arrows).

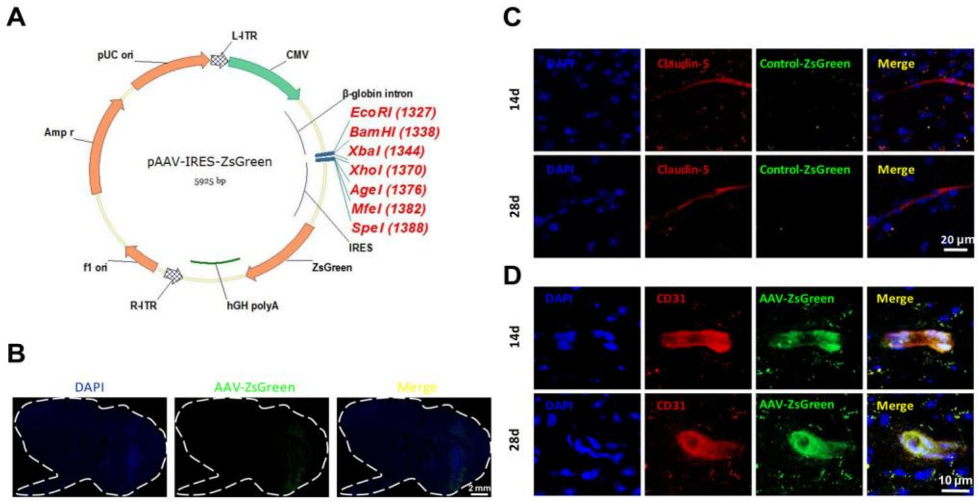

Figure S3. Enhanced Mfsd2a expression injected with Mfsd2a overexpression virus in perihematomas. (A)Schematic diagram of pAAV-Mfsd2a plasmid. Mfsd2a fragments were amplified by PCR and cloned into pAAV-IRES-ZsGreen vector by BamHI, EcoRI, Xbal, Xhol, Agel, Mfel, and Spel, to constructthe pAAV-Mfsd2a plasmid. (B) After two weeks, injection of Mfsd2a overexpression virus showed significant expression of AAV-ZsGreen. (C) Immunostaining for Control-ZsGreen (green), claudin-5-positive endothelial capillary profiles (red) in brain microvessels. The merged images of the overlay of ZsGreen together with claudin-5 are shown as yellow, and the nuclei are stained with DAPI (blue). Scale bar represents 20 μm. (D) Immunostaining for AAV-ZsGreen (green), CD31-positive endothelial capillary profiles (red) in brain microvessels from mice injected with Mfsd2a overexpression virus,The merged images of the overlay of AAV-ZsGreen together with claudin-5 were shown as yellow, and the nuclei were stained with DAPI (blue). Scale bar represents 10 μm.
